# Exome sequencing identifies a homozygous splice site variant in *RP1* as the underlying cause of autosomal recessive retinitis pigmentosa in a Pakistani family

**DOI:** 10.1080/07853890.2025.2470953

**Published:** 2025-03-03

**Authors:** Abdur Rashid, Asad Munir, Muhammad Zahid, Mukhtar Ullah, Atta Ur Rehman

**Affiliations:** a Department of Zoology, Islamia College University, Peshawar, Pakistan; b Department of Zoology, Faculty of Biological and Health Sciences, Hazara University, Mansehra, Pakistan; c Institute of Molecular and Clinical Ophthalmology Basel (IOB), Basel, Switzerland; d Department of Ophthalmology, University of Basel, Basel, Switzerland

**Keywords:** *RP1*, retinitis pigmentosa, splicing, consanguinity, Pakistan

## Abstract

**Background:**

Mutations in *RP1* gene are the third leading cause of inherited retinal dystrophies (IRDs) in Pakistani families.

**Patients:**

A two-generation consanguineous Pakistani family underwent both clinical and genetic analyses. Clinical examinations included visual acuity test, visual field, fundoscopy, and ocular coherence tomography (OCT). Whole exome sequencing (WES) was performed on the proband’s DNA, and Sanger sequencing was performed to validate the WES findings. Splicing prediction tools such as Human Splicing Finder (HSF), NNSplice predictor, SpliceAI, MaxENTScan, and SpliceRover were used.

**Results:**

A nuclear family of seven children, comprising five affected individuals (four males and one female) and two healthy siblings, was recruited from northwestern Pakistan. The proband was a 49-years old male who was presented with complaints of decreased visual acuity and night blindness since early childhood. Upon clinical evaluation, the proband appeared to have severely reduced visual acuity of hand movement (HM), bilateral visual field constriction, a waxy pale disc with vascular attenuation, pigmentary bone spicules at the periphery associated with chorioretinal degeneration, diffuse macular atrophy, and horizontal nystagmus in both of his eyes. Exome sequencing (ES) in the proband identified a homozygous splice site variant (NM_006269.2: c.615 + 1G > A) in *RP1* gene. *In-silico* analysis, genotype-phenotype co-segregation study, and literature survey strongly supported the causality of the detected variant.

**Conclusions:**

We report a previously known pathogenic splice site variant of *RP1* as the underlying cause of early-onset autosomal recessive retinitis pigmentosa (arRP) in a Pakistani family. We contemplate that the detected allele might constitute a mutational hotspot in *RP1*.

## Introduction

Retinitis pigmentosa (RP) is a genetically heterogeneous form of hereditary retinal dystrophies (HRDs) with a worldwide prevalence of 1 in 3000–4000 individuals [1]. Early signs of RP include night blindness or nyctalopia and visual field constriction or tunnel vision, which mostly appear in the first decade of life [2]. These symptoms are due to bilateral progressive degeneration of rod photoreceptor cells, which results in typical bone spicule pigmentation on the retina [2]. Later, cone degeneration starts, leading to variable loss of central vision and eventually, day blindness [3]. Globally, pathogenic variants in around 130 genes have been implicated in RP [RetNet; accessed June 5, 2024]. RP is the most prevalent form of HRDs in Pakistan with pathogenic variants identified in 37 distinct genes so far [4]. Following *PDE6A* and *TULP1* genes, *RP1* gene mutations are the third leading cause of HRDs in Pakistan [4]. Almost similar trend has been observed in the Arab countries where *RP1* and *TULP1* gene mutations are the frequent causes of rod-cone dystrophy [5].

*RP1* axonemal microtubule associated, formerly known as retinitis pigmentosa 1, is located on chromosome 8q11.23-q12.1, and is a photoreceptor-specific gene that was initially designated for autosomal dominant retinitis pigmentosa (adRP) [6]. The canonical *RP1* transcript (NM_006269.2) consists of four exons, including three coding exons, which translate into a polypeptide chain of 2156 amino acid residues with a molecular weight of 240 kDa [6]. In 2002, *RP1* protein was identified within human and mouse rod and cone photoreceptor cells connecting cilia and is believed to contribute to the transport of proteins between the inner and outer segments of photoreceptors or in maintaining ciliary structure [7]. Being localized in the photoreceptor axoneme, *RP1* regulates the length and stability of the axoneme by linking the outer segment discs to axonemal microtubules [7]. *RP1* has three domains, including two doublecortin (DCX) domains located between amino acid residues 28 and 228 and a putative domain located between amino acid residues 486 and 635. The DCX domain mediate interaction between *RP1* and microtubules and are primarily encoded by exons 2 and 3 [7,8].

Pathogenic variants of *RP1* are responsible for both autosomal dominant and autosomal recessive retinitis pigmentosa (ad/arRP) [9,10]. Globally, over 1100 distinct pathogenic variants of *RP1* have been reported in the ClinVar database. These variants predominantly comprised missense (*n* = 836), frameshift (*n* = 161), nonsense (*n* = 111), variants in UTRs (*n* = 8), and splice site variants (*n* = 6) [ClinVar; Accessed June 5, 2024]. Interestingly, protein-truncating variants located between amino acid residues 500 and 1053 of *RP1* exon 4 are associated with adRP, likely because of the dominant negative effect of the truncated RP1 protein [11,12]. However, pathogenic variants that cluster either toward the N- or C-terminus of *RP1* mostly result in arRP [13,14]. A recent review documented a total of 15 distinct *RP1* variants in Pakistani families with inherited retinal dystrophies (IRDs). These included 11 frameshifts, two missense mutations, one splice site, and one nonsense variant across 16 independent Pakistani RP families [4]. Here, we aimed to investigate the clinical and genetic characteristics of a consanguineous Pakistani family segregating autosomal arRP in multiple affected siblings. We hope that findings of our study may have implications for the native population. For instance, clinicians and/or scientists in the region may quickly screen their patients for the same allele, offer genetic counseling to the affected patients/families, recommend relevant therapies if feasible, and raise community awareness about the adverse clinical outcomes of consanguinity.

## Methods

### Ethics approval and clinical examination

The institutional review board of Hazara University Mansehra, Pakistan approved this study (approval code: F.No.185/HU/Zool/2022/583). This study adhered to the standards of the Declaration of Helsinki. Participants were well informed about the purpose of this study, and written informed consent was obtained for their participation as well as for publication of their medical history. Since all the participating patients were adults and no children were included in our study, they were all able to give informed consent by themselves. All patients were diagnosed with retinitis pigmentosa by an ophthalmologist following a through clinical examination. However, due to limitations in resources, a detailed clinical testing including visual field test, fundus photography, and OCT could be obtained for the proband only. In the proband, visual acuity was determined by illuminating a rotating Snellen chart. The type and amount of nystagmus were noted using the torch and prism tests. The cornea and anterior segment were examined using a slit lamp (SL.8Z Topcon; Japan). The posterior segment was examined using a direct ophthalmoscope (Welch Allyn, USA), indirect ophthalmoscope (Keeler, UK), and slit lamp (SL.8Z Topcon, Japan) with a 90 diopter (Volk, USA) biconvex lens. The macular and retinal nerve fiber layer thickness (presence and absence) was determined using an optical coherence tomography (OCT) machine (3D OCT-2000, Topcon, Japan). Fundus photographs were obtained using the California Ultra-widefield Retinal Imaging System (Optos California P200dTx), and the visual field was plotted using an automatic visual field analyzer (ZEISS-HUMPHREY, Germany).

### Sample collection and whole exome sequencing

Approximately 3 mL saliva samples were obtained from participants using a sterilized saliva self-collection kit (DNA Genotek Inc., Canada) following the manufacturer’s guidelines. Genomic DNA was extracted from saliva samples using standard protocols, as described in the MagMAX^™^ Saliva gDNA Isolation Kit manual (Thermo Fisher Scientific, USA). Quantitative assessment of DNA was performed using a NanoDrop 1000 spectrophotometer (Thermo Fisher Scientific, Waltham, MA, USA). For the qualitative assessment of DNA, 5 µL of the stock DNA sample was loaded onto a 1% agarose gel. Following gel electrophoresis at 120 V for 90 min, the DNA integrity was checked by visualizing the gel on a UV transilluminator.

Whole exome sequencing (WES) was performed by a commercial sequencing service (Novogene Co. Ltd, Cambridge, United Kingdom). For this purpose, approximately 2.0 μg of patient-derived genomic DNA was used as a template. Following manufacturers’ guidelines, the Agilent SureSelect Human All ExonV6 kit was used for capture of all coding part (exons) of the genome whereas HiSeq2500 (Novogene) was used for paired-end sequencing of the libraries. WES data were analyzed using a computational pipeline as described previously [15]. Briefly, raw sequencing reads were trimmed and high-quality sequencing reads were aligned against the hg19 (GRCh37) human reference genome using BWA [16]. Duplicate entries in the BAM files were identified using Picard while base quality score recalibrations were done using GATK [17]. Variants were called using GATK-HaplotypeCaller, and subsequently annotated with the help of ANNOVAR [18]. Using a strict filtration criterion, variants were prioritized according to their quality matrix, allele frequency (MAF ≤ 0.01), molecular impact (missense, nonsense, frameshift, and splicing effects) while keeping in mind the presumed pedigree-based inheritance patterns (AD, AR, XL). Finally, all shortlisted variants were checked using VariantValidator [19] for their correct genomic nomenclature. Several online prediction tools such as Human Splicing Finder [20], NNSplice predictor [21], SpliceAI [22], MaxENTScan [23], and SpliceRover [24] were used to predict potential effects of the identified intronic variant on mRNA splicing.

### Sanger sequencing and co-segregation analysis

Finally, the causality of the identified (pathogenic/likely pathogenic) variant was confirmed through familial genotype-phenotype co-segregation analysis using DNA from available family members. Briefly, a set of primers with forward (5′-GTCAACCCTCGCTCCTTTAAGT-3′) and reverse (5′-CACCATTCATATCCCACACG-3′) sequences that flanked our variant of interest was designed using the primer3 online tool for PCR amplification and subsequent Sanger sequencing of the target region. The PCR reaction was performed in a 20 µl volume using 2.0 ng of genomic DNA as a template. The PCR cycling conditions were 95 °C for 5 min for the initial denaturation, followed by 30 cycles of 95 °C for 30 s, 55 °C for 30 s, and 72 °C for 40 s. The final extension was performed at 72 °C for 5 min, and the reaction was maintained at 4 °C. The PCR products were visualized on a 2% agarose gel. The PCR product was purified using ExoSap-IT^TM^ and Sanger sequencing with a forward primer. Sanger sequencing files were visualized and compared with reference sequences using the CLC Genomics Workbench (QIAGEN). Finally, genome-wide homozygosity mapping was performed on exome data using default settings of the AutoMap [25] while individuals regions of homoygosity (ROHs) were ranked manually based on their genomic sizes available from the output files of AutoMap.

## Results

### Clinical findings

**Figure 1.**
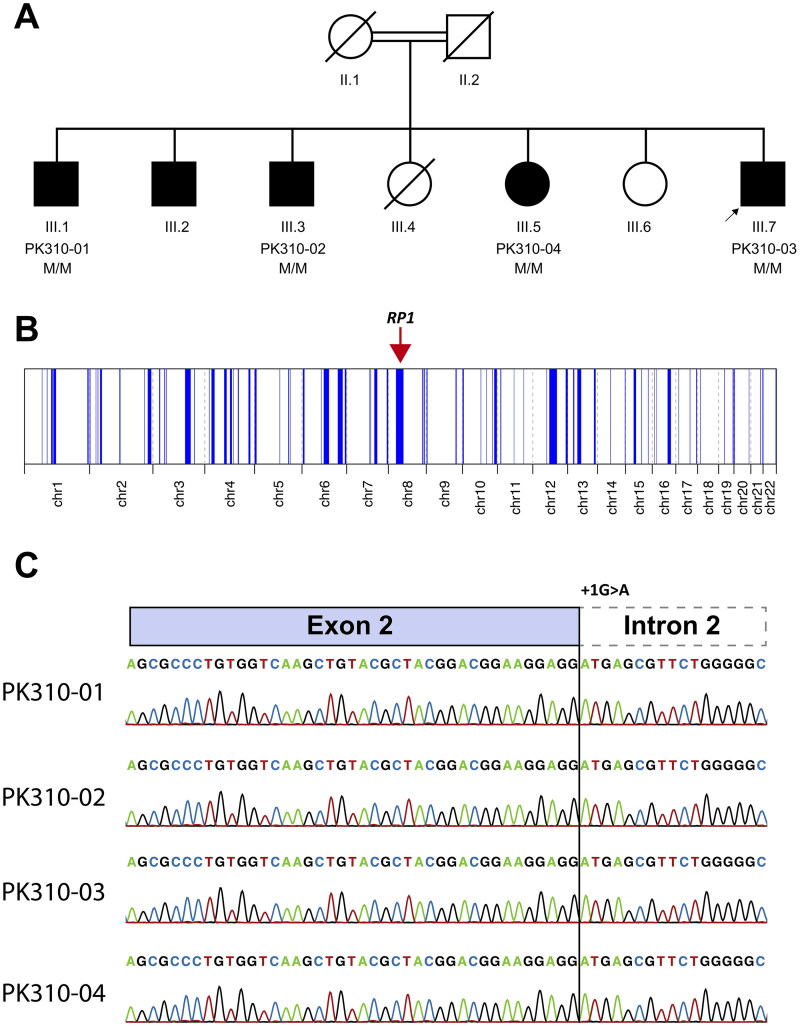
(A) Pedigree of the reported family showing affected individuals as dark-filled symbols while unaffected individuals as blank symbols. Double lines in first generation suggest parental consanguinity. Symbols with a diagonal line shows deceased individuals in the family. ‘M’ means identified mutation (c.615 + 1G > A) while a black arrow indicates the proband. (B) Homozygosity mapping showing chromosome-wise autozygous intervals as vertical blue peaks. A red arrow on top of chromosome 8 indicates region of homozygosity (ROH) harboring *RP1* gene. (C) Schematic representation of RP1 exon2-intron2 junction (top) while sanger electropherograms showing +1 G > a nucleotide change in the affected family members (bottom).

**Figure 2.**
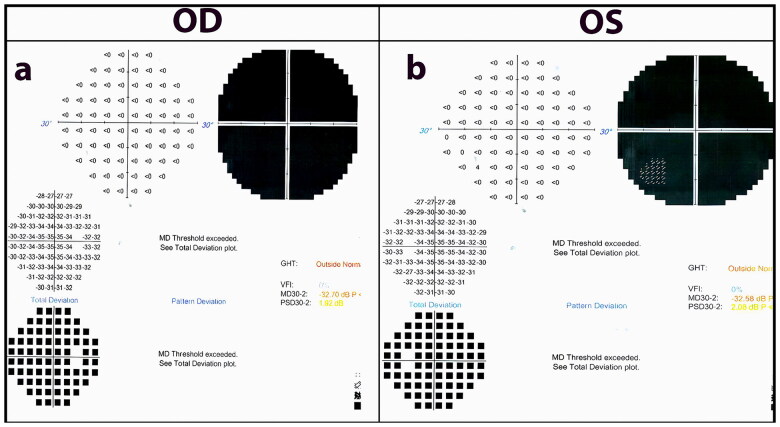
Bilateral visual field analysis test of *RP1* associated proband. Visual field analysis test (a) and (b) indicate generalized bilateral constriction of field.

**Figure 3.**
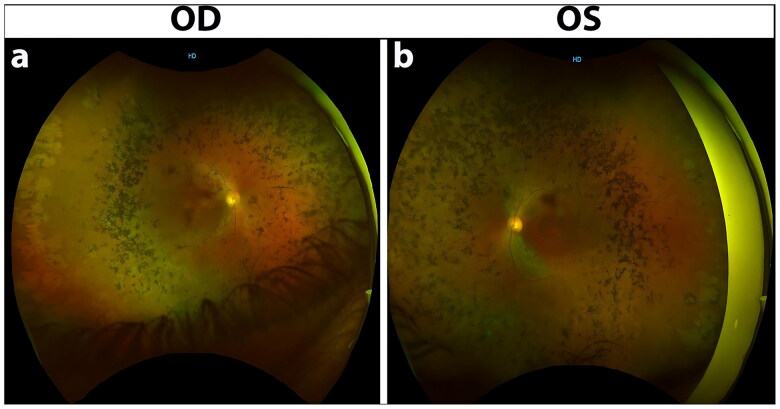
Fundoscopy images of patient with homozygous splice site *RP1* variant. Color fundus images (a) and (b) shows waxy pale disc, vascular attenuation and pigmentory bony spicule associated with chorioretinal degeneration.

**Figure 4.**
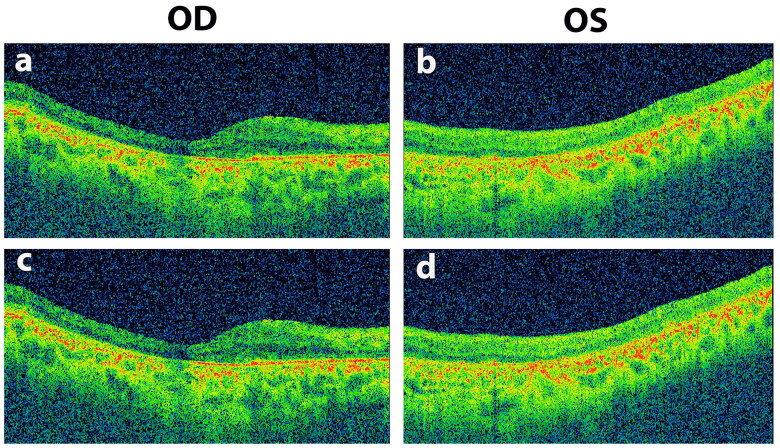
Optical coherence tomography macula (OCT-Macula) images of the proband (a), (b), (c) and (d) with homozygous splice site variant in *RP1*. OCT-Macula shows diffuse atrophy and thinning of the macula.

Two generations of a consanguineous family with multiple affected individuals were recruited from Mansehra district in northwestern Pakistan. The family consisted of seven children, including five affected individuals (four males and one female) and two unaffected females born to a consanguineous couple (Figure 1(A)). The proband was a 49-years old male who was presented with complaints of decreased visual acuity since early childhood. According to the proband, he developed night blindness 40 years ago, with a gradual loss of vision in both of his eyes. His medical and surgical histories were unremarkable; however, his family history was positive for Retinitis Pigmentosa (RP). The proband’s recent clinical re-evaluation revealed severely reduced visual acuity, which appeared to be of hand movement (HM), and had horizontal nystagmus in both of his eyes. The proband showed generalized bilateral constriction of the visual field, sparing a small area of vision in the center (Figure 2(a,b)). Fundus photography revealed a waxy pale disc with vascular attenuation and pigmentary bony spicule at the periphery associated with chorioretinal degeneration (Figure 3(a,b)). Optical coherence tomography macular (OCT-Macula) imaging of both eyes showed diffuse atrophy and thinning of the macula (Figure 4(a–d)).

### Genetic findings

Exome analysis of the proband revealed a single nucleotide intronic variant (c.615 + 1G > A) in *RP1* (NM_006269.2) in a homozygous state. Since the variant altered a highly conserved (PhyloP100 score = 9.692) intronic nucleotide at the canonical 5′ splice donor site (+1) at exon2-intron2 boundary, we assumed at first place that the identified variant is a likely pathogenic allele. To confirm this finding, a familial co-segregation study was conducted. The identified c.615 + 1G > A variant co-segregated with the disease as expected such that all available genotyped patients (*n* = 4) were homozygous for the putative allele (Figure 1(C)). The identified allele was not found in any major public databases, such as gnomAD and the 1000 Genomes Project. However, the identified c.615 + 1G > A variant was reported in the ClinVar database as ‘pathogenic’ in a German IRDs cohort. *In-silico* analysis using online splicing prediction tools such as Human Splicing Finder 2.4.1 (HSF), NNSplice predictor, SpliceAI, MaxENTScan, and SpliceRover, all predicted the variant to abolish the wild-type splice donor site. Furthermore, an accumulative 407.75 Mb (autosomal) genome was found to be autozygous in the proband, as reflected in the genome-wide homozygosity mapping. Interestingly, *RP1* gene was flagged inside an extended region of homozygosity (ROH) on chromosome 8. The ROH surrounding *RP1* gene spanned approximately 28.67-Mb in size, and was classified as the second largest ROH in terms of genomic length (Figure 1(B)). Thus, our homozygosity mapping results reaffirm parental consanguinity and an autosomal recessive inheritance pattern, as evident from the pedigree. Taken together, our results highlight the clinical relevance of the identified intronic variant (c.615 + 1G > A) in *RP1*.

## Discussion

Retinitis pigmentosa (RP) represents a subset of well characterized hereditary retinal diseases (HRDs) presenting both clinical and genetic heterogeneity [26]. Here, we report an intronic variant (c.615 + 1G > A) in *RP1* that, we believe, is the likely cause of arRP in five of the total seven siblings who were born to a consanguineous Pakistani couple. Previously, this variant was reported to be pathogenic in a German IRDs cohort [27]. Thus, we speculate that c.615 + 1G likely constitutes a mutational hotspot of *RP1*. In addition, we propose that the identified variant may cause the disease by disrupting the canonical donor splice site thus possibly leading to an aberrant mRNA splicing as predicted by several *in-silico* tools. We acknowledge here that our primary assertion about the pathogenicity of the detected variant was based mainly on genotype-phenotype correlation data, *in silico* analysis, and support from the literature. Thus, further studies are warranted to functionally validate the actual impact of the identified variant on the mRNA splicing. This is imperative because *in silico* tools alone have not yet been fully validated for use in clinical/definitive diagnosis [28].

Previously, 15 distinct *RP1* variants, including 11 frameshift, two missense, one nonsense and one splice site variant, were reported in Pakistani IRDs families [4]. Thus, our results slightly expanded the existing Pakistani *RP1* mutational repertoire to now 16 (likely) pathogenic variants. All previously reported *RP1* variants in Pakistani families were found in homozygous state and were correlated with arRP [9,29–32]. This closely aligns with our results, as we too found a homozygous variant segregating with arRP. The detection of *RP1* gene inside a large autozygous interval (28.67-Mb), and the presence of marked genome-wide total (autosomal) autozygosity (407.75-Mb) in the proband collectively reflects on the consanguineous nature of the Pakistani population. For instance, nearly half of the marriages in northwestern Pakistan are considered to be consanguineous [33–36] despite the fact that a recent study has shown a temporal decline in the rates of consanguinity in the region [37]. Thus, the utility of consanguinity-driven homozygosity mapping in the detection of disease-causing variants in rare Mendelian disorders remains promising [38,39]. Further­more, regardless of the nature or molecular impact of the detected *RP1* variants, all Pakistani *RP1*-associated patients were diagnosed with progressive (degenerative) arRP. Major clinical symptoms noted in published Pakistani *RP1*-related patients were optic disc pallor, bone spicule pigmentation, attenuated retinal arterioles, and maculopathy [29–32]. Although rare, blindness in the second decade of life was reported in three apparently unrelated RP1-associated Pakistani families [9]. Overall, these findings are comparable with those of our study, as we noticed a waxy pale disc, retinal vascular attenuation, bony spicule pigmentation with chorioretinal degeneration, generalized constriction of the visual field, and macular thinning in the proband. Specifically, our clinical synopsis supports an earlier study that documented severe maculopathy, bony spicule pigmentation, and retinal attenuation in a Pakistani pedigree associated with a bi-allelic splice site variant (c.787 + 1G > A) in *RP1* [29]. Moreover, studies have shown that pathogenic *RP1* variants are causative for both recessive and dominant RP [9,40]. For example, it has been ascertained that truncation of *RP1* protein before the bifocal gene product (BIF) motif or within the terminal portion will likely constitute a loss-of-function allele, thus resulting in arRP [31]. However, interruption of RP1 within or immediately after the BIF domain may result in a protein with a deleterious effect that causes autosomal dominant RP [31]. Unlike in the Pakistani population, *RP1* variants are believed to be the major cause of adRP in European cohorts [6,10,13,26,41,42]. It has also been postulated that most *RP1* mutations in European cohorts are protein-truncating alleles, and are mainly localized to a single hotspot region in *RP1* spanning between the c.1490 and c.3216 positions [42]. In addition, studies have shown that pathogenic variants in *RP1* exon 4 are generally causative for adRP, whereas pathogenic variants in *RP1* exon 2–4 are associated with arRP [11,12,14,40]. Lastly, heterozygous *RP1* mutations have often been found to cause mild RP phenotypes with late disease onset (usually by the third decade of life) while homozygous *RP1* mutations have been linked with a more severe phenotype and with variable disease onset [6,43–47]. Altogether, our findings are consistent with the published literature.

Currently, ∼30–40% of patients with IRDs still require an accurate molecular diagnosis despite the application of next-generation sequencing (NGS) methods [48]. Of the several reasons that can explain this missing heritability, one could be the presence of pathogenic variants in the non-coding regions (intergenic or intronic) of the genome [49]. The association of two intronic variants upstream of *TMEM216* gene with non-syndromic RP in patients of African and South Asian ancestry is only the tip of an iceberg [50]. Thus, keeping in view the unprecedented consanguinity rates in Pakistan, the odds of identifying additional deep intronic variants causing rare Mendelian diseases are very high. Furthermore, since the pace of new gene discovery rates in IRDs has nearly reached a plateau, scientists are now focused on the exploration of, once elusive or unrecognizable, DNA sequences such as copy number variations (CNVs), complex structural variants (SVs), and deep intronic variants with potential impacts on splicing [51,52]. This will not accelerate molecular diagnostic success rates by shrinking the existing missing heritability (∼30–40%) in IRDs but will also pave the way for the development of novel therapeutic options for IRDs. Since over 10% of total IRDs are linked to variants that alter the splicing machinery, the development of therapeutic interventions for correcting splicing defects is imperative. For instance, the use of antisense oligonucleotides (AONs), especially targeting complex splicing defects in *ABCA4*, has shown promising results in splicing modulation and disease correction in patients [53].

## Conclusions

We report a homozygous canonical splice site variant c.615 + 1G > A in *RP1* as the underlying cause of early onset RP in a Pakistani family with five affected children. Although this variant was previously reported in a German IRDs cohort, we are the first to report the same variant in a Pakistani pedigree. Due to the emergence of the same variant in two ethnically unmatched and geographically distant populations, we propose that the identified allele (c.615 + 1G > A) may constitute a mutational hotspot in *RP1*. Lastly, we hope that findings of our study may assist clinicians or scientists in the region to prescreen their patients in a time/cost-efficient manner such as Sanger sequencing. The patients/families may be offered genetic counseling and carrier testing to identify individuals at risk and to provide relevant therapies if feasible.

## Limitations of the study

Since our primary assertion about the pathogenicity of the identified intronic variant (c.615 + 1G > A) was based on genotype-phenotype correlation analysis, *in-silico* study and support from the literature, no data on the functional aspects of the identified allele were presented here. Thus, we emphasize the need for functional studies to fully understand the impact of the c.615 + 1G > A allele on the *RP1* mRNA splicing.

## Data Availability

All the data would be available from the corresponding author upon a reasonable request.
